# The shift of segmental contribution ratio in patients with herniated disc during cervical lateral bending

**DOI:** 10.1186/1471-2474-15-273

**Published:** 2014-08-12

**Authors:** Haw-Chang H Lan, Han-Yu Chen, Li-Chieh Kuo, Jia-Yuan You, Wei-Chun Li, Shyi-Kuen Wu

**Affiliations:** Department of Physical Therapy, HungKuang University, No. 1018, Sec. 6, Taiwan Boulevard, Taichung, Shalu District 43302 Taiwan; Department of Radiology, Taichung Veterans General Hospital, Taichung, Taiwan; School of Radiological Technology, Central Taiwan University of Science and Technology, Taichung, Taiwan; Department of Occupational Therapy, National Cheng Kung University, Tainan, Taiwan; Department of Physical Therapy, I-Shou University, Kaohsiung, Taiwan

**Keywords:** Cervical spine, Intervertebral motion, Segmental contribution, Biomechanics

## Abstract

**Background:**

Abnormal intervertebral movements of spine have been reported to be associated with trauma and pathological conditions. The importance of objective spinal motion imaging assessment in the frontal plane was frequently underestimated. The clinical evaluation of the segmental motion contribution could be useful for detecting the motion pattern of individual vertebrae. Therefore the purpose of this study was to investigate the shift of segmental contribution ratio in patients with herniated disc during cervical lateral bending to provide additional insights to cervical biomechanics.

**Methods:**

A total of 92 subjects (46 healthy adult subjects and 46 disc-herniated patients) were enrolled in this case–control study. The motion images during cervical lateral bending movements were digitized using a precise image protocol to analyze the intervertebral motion and contribution.

**Results:**

Our results showed that the intervertebral angulation during cervical lateral bending for the C2/3 to C6/7 segments were 7.66°±2.37°, 8.37°±2.11°, 8.91°±3.22°, 7.19°±2.29°, 6.31°±2.11°, respectively for the healthy subjects. For the patients with herniated disc, the intervertebral angulation for the C2/3 to C6/7 segments were 6.87°±1.67°, 7.83°±1.79°, 7.73°±2.71°, 5.13°±2.05°, 4.80°±1.93°, respectively. There were significant angulation and translational differences between healthy subjects and the patients with herniated disc in the C5/6 and C6/7 segments (P=0.001-0.029). The segmental contributions of the individual vertebral segments were further analyzed. There was a significant increase in segmental contribution ratio of C3/4 (P=0.048), while a significant decrease in contribution ratio of C5/6 (P=0.037) was observed in the patients with herniated disc. Our results indicated that the segmental contribution shifted toward the middle cervical spine in the patients with herniated disc.

**Conclusions:**

The segmental contributions of cervical spine during lateral bending movement were first described based on the validated radiographic protocol. The detection of the shift of segmental contribution ratio could be helpful for the diagnosis the motion abnormality resulted from the disc or, facet pathologies, and arthritic changes of cervical spine.

## Background

The cervical spine is to contain and protect the spinal cord, support the skull, and enable diverse neck movement. Cervical disorders alter the neck’s normal active range of motion that is a useful parameter to determine the level of function and to establish a treatment plan, monitor the patient’s progress and the effectiveness of therapeutic interventions [1–4]. The intervertebral disc serves as a strong, flexible interface between adjacent vertebral bodies, and is responsible for transmitting loads in different directions while permitting movements of spinal column [1, 2]. A herniated disc sometimes leads to the irritation of spinal nerves and can cause neck/back pain and motion dysfunction [3]. Clinically, the physical examination of the disc-herniated spine often reveals a decrease in the spinal motion range in the affected area [3, 4]. Without resorting to invasive techniques, the precise assessment of clinically relevant variables, such as intervertebral movements, is difficult to obtain. Several studies were conducted to explore the sagittal plane intervertebral angles or spinal curvature by plain radiographs [5–8]. Sohn and colleagues [9] investigated the relationships between disc degeneration and vertebral morphologic changes in cervical spine. Their findings indicated that the increased disc bulging was correlated with the decreased segmental angles in the sagittal plane; however, the correlation to the frontal plane motion was not addressed. Giuliano et al. [10] also proposed that the cervical disc herniation were associated with the motion restriction and change of lordosis curvature in sagittal plane. The abnormal or excessive motions between vertebrae in the both sagittal and frontal planes are clinically important clues to dysfunction or instability [7–11]. Harrison et al. [12] had pointed that the spinal movement in the frontal plane was largely neglected in the biomedical literatures. Janik and relations [13] also reported that the evaluation of segmental dysfunction desired the quantification of the lateral bending movement in the antero-posterior view of radiographs.

A few researches have indicated that the highly repetitive flexion and lateral bending motions resulted in intervertebral disc herniation [14, 15]. Costi et al. [2] further proposed that the maximum physiological range producing the highest physiological shear strain were the lateral bending and flexion, followed by lateral shear and compression. Consequently, the lateral bending movement in the frontal plane of spinal motion segments could possibly placed the disc at greater risk, in addition to the flexion maneuver. The cervical motion measures provide substantial information regarding the severity of function and motion limitation. The lumbar disc herniation has sometimes been reported to lead to the musculoskeletal findings of acute tilt or impaired lateral mobility to one side or the other. The level of disc herniation in lumbar spine was suggested to be determined by lateral bending roentgenograms on the frontal plane [16]. Clinically, the spinal mobilization technique has been frequently applied in the lateral directions to restore the normal intervertebral motion and to open the intervertebral foramen in the treatment of patients with spinal pain [17–19]. The importance of an objective spinal motion imaging assessment was emphasized in the sagittal and frontal motion planes, though the lateral bending motion of cervical spine was rarely investigated [20, 21].

On the other hand, the movements of a normal cervical spine can be achieved only with the contribution of each cervical functional segment. Abbott and colleagues [22] have introduced an approach to diagnosing lumbar segmental mobility disorders by a normalized within-subjects contribution to total-motion model, which was intended to identify segments contributing significantly more, or significantly less, to total lumbar motion. The benefit of normalized within-subjects contribution to total-motion approach was sensitive for defining lumbar segmental mobility disorders. Miyazaki et al. [23] reported that the changes in the sagittal alignment of the cervical spine might affect the kinematics and the contribution of each segment to the total angular mobility. Consequently, it may cause changes in the segment subjected to maximum load for overall motion and accelerate its degeneration. The simulated restricted neck range of motion has documented to affect the percentage contribution among the spinal levels and demonstrated unusual motion patterns from those of the normal subjects [24]. Dvir and associates [25] further considered the segmental and total cervical range of motion as a suitable parameter for the interpretation of cervical motion limitations in neck patients. Most patients with disc herniation may have a certain degree of abnormal spinal flexibility; however, the relationship between spinal kinematics and disc herniation has not been fully investigated. The aim of this study was to investigate the segmental contribution among the cervical spine levels between healthy subjects and patients with herniated disc by a standard radiographic image protocol. The information could be helpful for the diagnosis the motion abnormality resulted from the disc herniation of cervical spine.

## Methods

A total of 92 subjects (46 healthy adult subjects and 46 disc-herniated patients) participated in this case–control study. The healthy subjects have no neck symptoms within recent 4 weeks and were excluded if she/he had (1) history of cervical trauma or surgery, (2) bone pathology, (3) arthritic or other inflammatory disorders, (4) pregnancy, and (5) restrictive muscle spasm. Forty-six diagnosed C4/5 and/or C5/6 disc-herniated patients with neck, shoulder blades or radiating arm symptoms (such as: pain, sensory disorder, reflex abnormalities, and motor weakness) within recent 3 months were recruited from the Department of the Physical Medicine and Rehabilitation in a medical center to enroll in this study. The patients were excluded if she/he had (1) history of cervical surgery, such as disc replacement, bone fusion, and discectomy, (2) significant potential for cord injury, such as cord impingement from a large disk herniation, (3) advanced cervical spondylosis, (4) severe spinal stenosis, (5) inflammatory arthritic disorders (ankylosing spondylitis, rheumatoid arthritis), (6) severe spinal instability, and (7) pregnancy [6, 10, 18]. The age of the participants ranged from 20 to 45 years and clinical characteristics of the patients were documented. This study was approved by the Ethical Committee in Human Research of the Taichung Veterans General Hospital, TAIWAN. The experimental procedures and risks of the radiation exposure were fully explained to each patient, and a signed informed consent was obtained.

The videofluoroscopy system (Diagnost 97, Philips Corporation, USA) was applied to evaluate the continuous segmental movement of the cervical spine at a rate of 30 frames per second. The radiographic beam field of the videofluoroscopy unit was collimated to obtain optimal sharpness of the image. The size of the imaging field was also adjusted to view the whole lateral bending movements of cervical spine. The subjects placed their forearms on the armrests aside the examination table to reduce excessive trunk movement. Before actual screening, the subjects practiced the right and left lateral bending movements of the cervical spine a few times with correction to reduce the trunk and out-of-plane motions. Though the fluoroscopic image sequences could provide an objective and precise quantification of intervertebral movement, the out-of-plane motion and errors in reference point placement should be considered [20, 26]. To increase accuracy of intervertebral translation measurement in 2-D representation of the videofluoroscopy system, it is important to align the projection direction of the radiographic beam perpendicular to the plane of movement. Two portable laser alignment devices were applied to assure the perpendicularity between the motion plane of cervical movement and the projection direction of the radiographic beam. A semicircular guide was also positioned in the frontal plane by side of the subjects during cervical lateral bending motion. The whole motion sequences of the global motion of the cervical lateral bendings were captured real-time by a digital camcorder (Handycam HDR-PJ30, Sony Inc., Japan) to guide the subjects to move in the correct frontal motion planes. The subjects could visualize his/her neck motion by the display of the widescreen LCD throughout the lateral bending movements.

The subjects were instructed to move at a modest, constant rate to avoid the motion blur or excessive radiation exposure. The lateral bending of cervical spine from the neutral position to the right side, to the left side and return to the neutral position in five seconds with mouth open and closed conditions were performed in order to have clear images of the upper and lower cervical vertebra bodies, respectively. The recorded video images of the cervical motion were captured at 30 frames/second using the Avid image capture system (Avid Corporation, USA) and then transformed into the sequences of bitmap pictures. Three pictures in neutral position and the end of right and left lateral bending movements were selected for digitizing respectively. During the image analysis procedure, the positions of the 22 bony landmarks were digitized utilizing SigmaScan 5.0 (SPSS Inc., Chicago, IL, USA) on a high resolution monitor. The anatomical identifications of the bony landmarks were based on the well-accepted radiographic method of Frobin et al. [7, 8, 20, 26]. They were two inferior corners of the second vertebra (C2), and the anterior- posterior corners of the superior and inferior endplates from the third to seventh cervical vertebrae (C3-C7). The methods for identifying vertebral landmarks were blinded between examiners and totally two sets of 276 image pictures (92 subjects × 3 images) were digitized by two spinal research staffs. Three petitions of digitizing and their mean values were used for subsequent analysis. The width of next upper vertebral endplate was used to normalize the measurement of intervertebral movements during cervical lateral bending. This method of skeletal landmarks identification has been proved valid, accurate, and reliable for detecting the vertebral movements [2, 12, 26].

**Figure 1:**
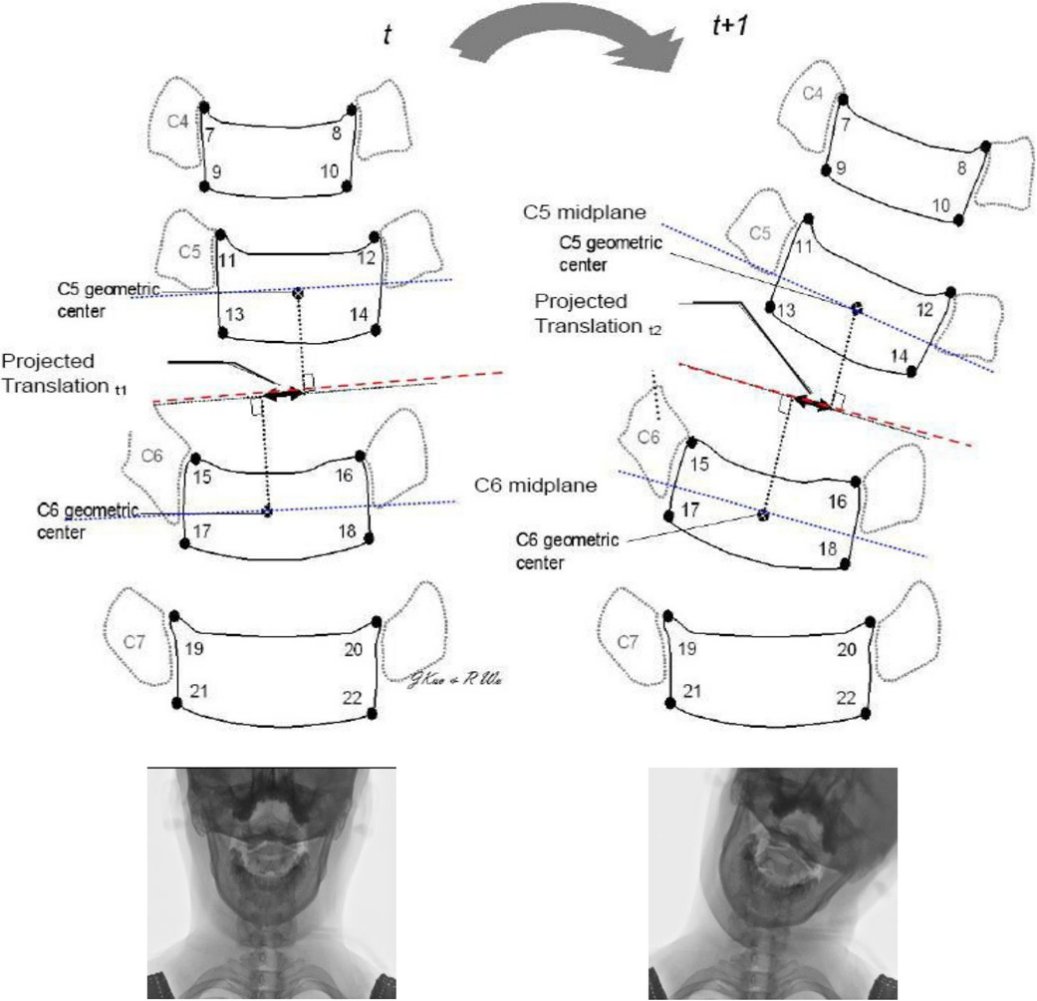
**The identifications of bony landmarks and intervertebral angulation and translation during lateral bending movements.**

A custom computer program was used to construct the midplanes of vertebrae defined as a line running through the midpoints between anterior two corners and posterior two corners, bisectrix between two midplanes, and the perpendiculars from centers of the adjacent vertebrae in order to calculate the relative angulation and translation of cervical spine [8, 12] (Figure 1). The segmental angulation change was calculated by the bisectrix between two midplanes. The width of adjacent upper vertebral endplate was adopted to normalize the measurement of intervertebral translation during lateral bending movement. The segmental contribution of each level to the total angular mobility of the cervical spine during lateral bending was defined as percentage segmental mobility, which was calculated as follows [22, 23, 25]: (angular variation of each segment in degrees)/(total angular motion in degrees) × 100.

The reliabilities of the digitizing procedures within raters at a 2-weeks interval and between raters were assessed with the intraclass correlation coefficient (ICC) and mean absolute difference (MAD) methods [8, 9, 26]. Comparisons among intervertebral movements were performed with one-way analysis of variance (ANOVA) with a probability level of *P* < 0.05 selected as the criterion for noting significant difference. For any statistically significant findings obtained in the ANOVA, the Tukey’s studentized range (HSD) test was performed to identify the differences among 5 vertebral levels in lateral bending movements. For the comparison at the same spinal level, a student t-test with a probability level of *P* < 0.05 was selected as the criterion for noting significant difference of segmental contribution between healthy subjects and patients with herniated disc. Analyses were performed using the Scientific Package for Social Sciences (version 13; SPSS, Chicago, IL, USA). We confirm that our research has adhered to the STROBE guidelines.

## Results

A total of 92 subjects participated in this study and the mean ages of the 46 healthy participants were 28.7 ± 5.4 years (21 females and 25 males). The body heights and weights were 161.4 ± 4.9 cm and 54.9 ± 4.6 kgw for the female subjects, and 171.7 ± 6.2 cm and 74.5 ± 6.6 kgw for the male ones. The mean ages of the 46 patients with herniated disc were 31.5 ± 6.4 years (24 females and 22 males). Their body heights and weights were 160.2 ± 5.6 cm and 54.4 ± 6.2 kgw for the female subjects, and 174.1 ± 5.2 cm and 78.4 ± 7.2 kgw for the male ones.

### Evaluation of errors and repeatability

The test-retest reliabilities of the digitizing procedures within raters were examined at 2-weeks interval. The ICCs for the calculated angular movements throughout the lateral bending averaged 0.824 and 0.974. The corresponding MAD averaged 2.36° ± 0.84° within raters. The ICCs for the calculated translation movements throughout the lateral bending ranged from 0.728 to 0.922 (mean = 0.836). The corresponding MAD averaged 0.26 mm ± 0.14 mm within raters.

Considering the inter-rater reliability, the ICC values of angulation averaged 0.875 with a MAD of 2.52° ± 0.92°. The ICC values for the calculated translation movements ranged from 0.718 to 0.911 (mean = 0.819) and the MAD averaged 0.30 mm ± 0.19 mm between raters. The digitization of image process demonstrated the good reliabilities within and between raters.

### Intervertebral angulation and translation

**Figure 2:**
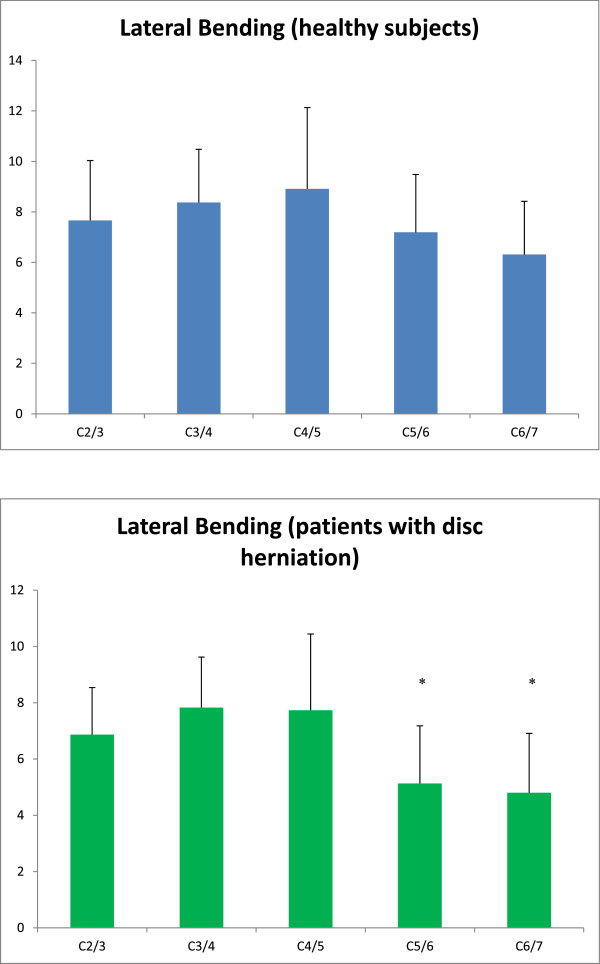
**Intervertebral angulation for the C2/3 to C6/7 segments during lateral bending movement for the healthy subjects and the patients with herniated disc.**

The intervertebral angulation for the C2/3 to C6/7 segments were 7.66° ± 2.37°, 8.37° ± 2.11°, 8.91° ± 3.22°, 7.19° ± 2.29°, 6.31° ± 2.11°, respectively for the healthy subjects. The Tukey HSD tests showed the significant difference between C3/4 and C6/7; C4/5 and C5/6; C4/5 and C6/7 levels (P < 0.05) (Figure 2). On the other hand, there were 46 patients with herniated disc enrolled in this study. The intervertebral angulation for the C2/3 to C6/7 segments were 6.87° ± 1.67°, 7.83° ± 1.79°, 7.73° ± 2.71°, 5.13° ± 2.05°, 4.80° ± 1.93°, respectively for the patients. The Tukey HSD tests showed the significant differences between C2/3 and C5/6, C6/7 levels; between C3/4 and C5/6, C6/7 levels; between C4/5 and C5/6, C6/7 levels (P < 0.05) (Figure 2).

**Figure 3:**
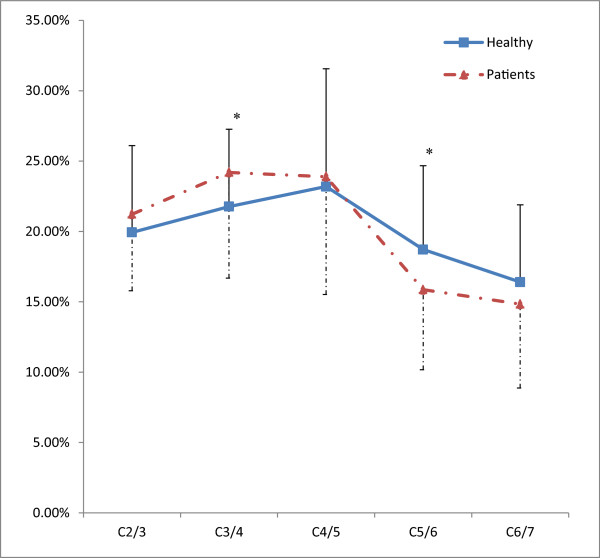
**The comparison of segmental contribution ratio during the lateral bending movement between healthy subjects and the patients with herniated disc.**

For the comparisons between healthy subjects and the patients with herniated disc, the intervertebral angulation of the individual segments during lateral bending significantly decreased in the C5/6 and C6/7 segments (P = 0.001) for the patient group. The segmental contribution ratio of the individual vertebral segments during lateral bending movement for the healthy subjects and the patients with herniated disc were separately presented in Figure 3. There was a significant increase in the segmental contribution of C3/4 (P = 0.048), while a significant decrease in the segmental contribution of C5/6 (P = 0.037) was observed in the patients with herniated disc (Figure 3). The segmental contribution ratio of the patients with herniated disc during lateral bending movement seemed to shift toward the middle cervical spine when comparing with those of healthy subjects. For the comparisons between healthy subjects and the patients with herniated disc, the intervertebral translations of the individual vertebral segments during the right and left lateral bending were summarized (Table 1). There were significant differences between healthy subjects and the patients with herniated disc in the C5/6 and C6/7 segments (P = 0.001-0.029).

**Table 1:** **The intervertebral translation of the individual vertebral segments during the right and left lateral bending for the healthy subjects and the patients with herniated disc**

		Healthy				Patient				Significance (2-tailed)
		Mean	SD	95% Confidence interval		Mean	SD	95% Confidence interval		
				Lower	Upper			Lower	Upper	
				Bound	Bound			Bound	Bound	
Right lateral bending	C2/3	1.24	0.39	1.12	1.36	1.25	0.48	1.10	1.40	0.914
	C3/4	1.58	0.46	1.44	1.73	1.66	0.55	1.48	1.83	0.516
	C4/5	1.96	0.63	1.76	2.15	1.80	0.61	1.61	1.99	0.237
	C5/6	1.76	0.53	1.59	1.92	1.07	0.38	0.96	1.19	0.001^*^
	C6/7	0.89	0.79	0.64	1.13	0.57	0.49	0.41	0.72	0.029^*^
Left lateral bending	C2/3	1.20	0.44	1.07	1.34	1.17	0.40	1.05	1.29	0.689
	C3/4	1.61	0.47	1.46	1.76	1.58	0.55	1.41	1.76	0.628
	C4/5	1.90	0.64	1.70	2.10	1.87	0.65	1.67	2.07	0.763
	C5/6	1.66	0.60	1.47	1.85	1.21	0.42	1.08	1.34	0.001^*^
	C6/7	1.01	0.73	0.78	1.24	0.73	0.44	0.59	0.87	0.028^*^

^*^ *P* < 0.05 compared between healthy subjects and patients with herniated disc.

## Discussion

This study characterized the angular and translational movement between healthy subjects and patients with herniated disc during cervical lateral bending in vivo application. There was a significant increase in segmental contribution ratio of C3/4, while a significant decrease in contribution ratio of C5/6 was observed in the patients with herniated disc. Our results indicated that the segmental contribution shifted toward the middle cervical spine in the patients with herniated disc.

In general, our mean values and standard deviations of each intervertebral angulation and the trend of motion contribution among segments were similar to the findings of previously published studies [21, 27, 28]. Ishii et al. [28] and Panjabi et al. [29] reported that the mean lateral bending at each level of the cervical spine to one side ranged 3.3° to 5.7° and 2.7° to 4.8°, respectively. In the present study, the subjects performed the right and left lateral bending movements, and the total range of lateral bending were documented as the outcome measurements (Figure 2). Their lateral bending ranges were about the half amount of the angulation findings in our healthy subjects. Panjabi and associates [29] investigated the mechanical properties of multilevel human cervical spines by applying rotational moments to specimens. The load–displacement curves revealed the ranges of motion measured from lateral bending were 5.4° - 9.6°. Their total angulations of C5/6 and C6/7 levels were further found to be significantly smaller than other cervical segments (C2/3-C4/5). The similar scenario of motion contribution among segments was also observed in the present study.

Spinal motion generally decreased if a disc herniation has occurred. Most patients with disc herniation may have a certain degree of abnormal spinal flexibility; however, the relationship between kinematics and herniation has not been investigated fully. The cervical range of motion device had been applied for measuring lateral flexion in patients with neck pain [30]. Though the lateral flexion ranges in patients with neck pain were reported to be lesser than those in the normal adults, the precise assessment of clinically relevant variables, such as intervertebral movements, is difficult to obtain. Disc degeneration or herniation is usually believed to induce structural changes within the disc that ultimately result in decreased disc height. Though the radiographic evaluation was feasible for the assessment of disc degeneration, the motion profiles of the spine with herniated disc were not addressed [31]. The patients with herniated disc significantly decreased the angulation ranges on C5/6 and C6/7 motion segments when compared to those of healthy subjects in the present study. The disc heights had proposed to have a proportionally linear relationship with the sagittal plane angle. The loss of disc height was regarded as one sign of clinical vertebral degeneration and the restoration of disc height was documented to have a positive effect on spinal range of motion [7, 32]. Weitz [16] had described that the lumbar disc herniation could lead to the musculoskeletal findings of acute tilt or impaired lateral mobility to one side or the other. These findings of reduced motion on the lower cervical spine may reflect a protective mechanism to splint the affected disc space in the position where the disc prolapsed exerting the least possible pressure on the affected nerve root. Daffner and colleagues [33] investigated the mechanical properties of multilevel human cervical spines by applying pure rotational moments to each specimen. They further reported that cervical disc herniation resulted in a decrease in angular motion of 2.8% - 5.2% per mm herniation at levels adjacent to the herniation. The scenario of reduced spinal mobility of C5/6 and C6/7 levels in patients with herniated disc needed more careful interpretation. With the older age of the herniated disc group in the present study, there may be a decrease in range of motion [34]. Moreover, the lower segment in cervical spine the segmental angular motion decreased. The role of the uncovertebral joints may play an important role in the regulation of primary lateral bending movement and the lateral degenerative processes may influence both primary and coupled movements [35]. This is further evident by comparing percentage angular motion for the two groups and C6/7 level is not statistically different.

Although the total ranges of motion in cervical spine were usually studied to provide the global function of the neck, it did not reveal what actually happened at the segmental levels. Abbott et al. [22] have introduced a segmental contribution approach to evaluate lumbar segmental instability, which intended to identify segments contributing significantly more, or significantly less, to total lumbar motion, compared to other segments within the same individual. They provided the reference intervals for the sagittal rotation and translation for estimating prevalence of lumbar segmental instability population. A recent study has quantitatively measured the percentage contribution of segmental angular motion during different motion ranges of cervical flexion-extension for healthy subjects [36]. Their findings indicated that the cervical flexion movement initially relied more on the middle cervical spines and later on the lower ones; whereas a different motion pattern trend from lower to middle segments was observed during cervical extension. The evidence regarding the segmental contribution ratio during cervical lateral bending was first investigated for the comparison between healthy subjects and patients with herniated disc in the present study. The cervical lateral bending movement relied more on the C3/4, C4/5, and C5/6 levels in our healthy subjects. Miyazaki and associates [23] examined the degenerated changes in the cervical disc and its relationship to the extent of cervical spine mobility. Their percentage contribution ratio of angular motion were also reported to be greater in C4/5 and C5/6 motion segments in the subjects with grade I disc degeneration, which grade definition still had normal intervertebral disc height with clear distinction of nucleus and annulus.

There was a significant increase in the percentage contribution ratio of C3/4, whereas a significant decrease in the percentage contribution ratio of C5/6 was observed in the patients with herniated disc (Figure 3). Interestingly, the segmental contribution ratio statistically shifted from C5/6 to C3/4 level in our patients with disc herniation. This finding might imply that lower cervical painful symptoms possibly lead to the condition that a trend of motion pattern shift from the lower to middle cervical segments (C3/4 and C4/5) in the disc-herniated patients. The reduced segmental contribution on the lower cervical spine of disc-herniated patients may reflect a protective mechanism to perform a neck movement that the prolapsed disc exerting the least possible pressure on the affected mobile segment [16]. The loss of normal disc height or lordotic alignment resulted from disc herniation or degeneration was documented to induce pathologic changes in the spinal kinematics and may accelerate degeneration of the functional motion unit of spine [23]. Daffner and colleagues [33] have performed the measurements of disc herniation for stability evaluation, which included static intervertebral angular displacements and translations in flexion, neutral, and extension as well as the intervertebral disc height. However, in an attempt to stabilize joint or ligament laxity, slight increases in angular motion occurred in other nonadjacent segments, although the total increased motion from these nonadjacent segments was not as great as the decreased motion associated with herniated segments. Miyazaki et al. [23] also reported the similar fashion that the angular range of motion decreased at all levels, the percent contribution ratios to total angular range of the C2/3 and C3/4 levels tended to increase in the disc degeneration condition. In contrast, the percent contribution ratios to total angular range of the C4/5, C5/6 and C6/7 levels tended to decrease. Though the disc herniation and degeneration might have some different clinical characteristics of disc property changes, there is a close cause-and-effect relationship between each other. Degenerative changes in the cervical spine are an inevitable response to the aging process. With degeneration, the disc can sometimes herniated through the surrounding outer annulus fibrosus and irritate adjacent nervous tissue. On the other hand, a sudden injury leading to a herniated disc (such as a fall) may also begin the degeneration process [9, 14, 15, 33]. The segmental contribution ratio between cervical segments might be useful in elucidating the relationship between disc herniation within the cervical spine and their impact on the angular motion, despite the individual variations among subjects.

There was little information about the vertebral shear or translation results in the frontal plane form the earlier researches. The intact cervical segment permitted a maximum of 3.5 mm translation before the removal of surrounding ligaments and facet joints. The translation movement between vertebrae greater than 3.5 mm or 20% of the vertebral width was suggested to be an indicator of spinal instability or pathologies [8, 11, 22]. The average measured translation to the right and left lateral bending in the present study ranging from 0.57 mm to 1.96 mm appeared to be within the reasonable range of translation in the cervical spine, however, the measurement of the intervertebral translation based on the single observation of a range of motion must be interpreted carefully. After adjusted for the normalized width of individual vertebrae, our results of translation percentages relative to the next adjacent vertebrae were 2.7% to 9.4%. For the comparisons between healthy subjects and the patients with herniated disc, the patient group significantly decreased the translation motion in the C5/6 and C6/7 segments compared to the healthy subjects. Daffner et al. [33] controlled the degree of disc degeneration, age, and gender, to measure the effect of disc herniation. They reported an average of 7.2% decrease in translation motion per mm of disc herniation at the levels above disc herniation but the levels below the disc herniation did not experience any significant change in motion. However, the translation movement adjacent to a lower cervical spine disc herniation significantly decreased 12.7% - 25.6% as disc degeneration progressed. Their research findings may help to support the results of the decreased translation motion on the lower cervical spine in the present study. Hussain and associates [37] had developed a poroelastic, three-dimensional finite element model of a normal C5/6 segment. A decrease in segmental flexibility was also reported to be associated with disc degeneration and the biomechanical effect of degenerative disc changes on the disc pressure was higher in lateral bending condition.

The measurement of the kinematics in the frontal plane in this study has some limitations. The voluntary lateral bending has been reported to be accompanied by limited flexion-extension or axial rotation [30, 38]. It has been identified that two-dimensional analysis of coupling motions may not fully report the accurate axial rotation movement [38, 39]. Yoganandan et al. [34] investigated the range of motion of axial component from lateral flexion tests in cervical cadaveric spine. Their results indicated that while the greatest axial ranges of motion occurring from C3 to C5 levels showed cranial and caudal decreases. In contrast, Ishii et al. [28] proposed that coupled axial rotation to lateral bending movement was observed as 0.8-1.8 degrees in the sub-axial cervical levels. The coupled flexion-extension motion was small at all vertebral levels (<1.1°). Though their study suggested that the clinical utility of coupling may be limited during diagnosis, the small coupling motions may not be ignored for clinical clinicians. The quantitative analysis of the segmental contribution may be employed to diagnose movement abnormalities like hypomobility or hypermobility and to monitor the treatment effect on the cervical spines; however, our study focused on two groups of similar age in healthy subjects and patients with disc herniation. Future researches expand the subject and patient groups across different spinal problems and ages may reveal more complicated or even compensatory movements for the spinal impairment.

## Conclusion

To summarize, the intervertebral translations of cervical spine during right and left lateral bending were described based on the validated radiographic protocol. With the advantages of visualization of vertebral segments, the reliable image technique is considered feasible in clinical and research applications. The segmental contribution ratio statistically shifted from C5/6 to C3/4 level in our patients with disc herniation. This finding might imply that lower cervical painful symptoms possibly lead to the condition that a trend of motion pattern shift from lower to middle cervical segments (C3/4 and C4/5) in the disc-herniated patients. The detection of the shift of segmental contribution ratio could be helpful for the diagnosis the motion abnormality resulted from the disc or, facet pathologies, and arthritic changes of cervical spine.
